# 5-Cyclo­pentyl-2-(4-methyl­phen­yl)-3-methyl­sulfinyl-1-benzofuran

**DOI:** 10.1107/S1600536812042249

**Published:** 2012-10-13

**Authors:** Hong Dae Choi, Pil Ja Seo, Uk Lee

**Affiliations:** a Department of Chemistry, Dongeui University, San 24 Kaya-dong, Busanjin-gu, Busan 614-714, Republic of Korea; b Department of Chemistry, Pukyong National University, 599-1 Daeyeon 3-dong, Nam-gu, Busan 608-737, Republic of Korea

## Abstract

In the title compound, C_21_H_22_O_2_S, the cyclo­pentyl ring adopts an envelope conformation with the flap atom connected to the benzofuran residue. The benzofuran unit is essentially planar, with a mean deviation from the least-squares plane defined by the nine constituent ring atoms of 0.008 (2) Å. In the crystal, mol­ecules are linked *via* pairs of C—H⋯π inter­actions, forming inversion dimers. In the ring of the 4-methyl­phenyl group, four C atoms and their attached H atoms are disordered over two sets of sites, with site-ccupancy factors of 0.899 (5) and 0.10.

## Related literature

For background information and the crystal structures of related compounds, see: Choi *et al.* (2011 ▶); Seo, *et al.* (2011 ▶).
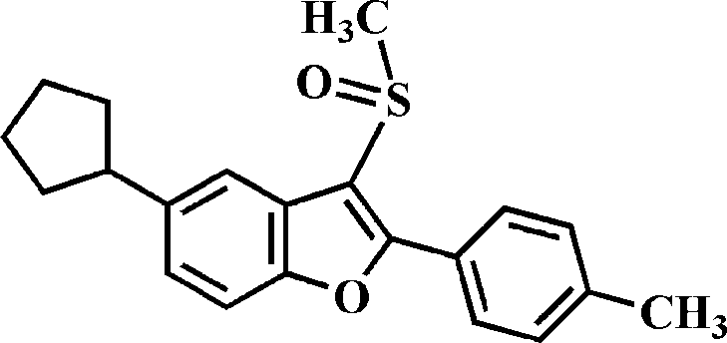


## Experimental

### 

#### Crystal data

C_21_H_22_O_2_S
*M* *_r_* = 338.45
Monoclinic, 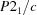

*a* = 15.2869 (5) Å
*b* = 7.2881 (3) Å
*c* = 15.3608 (6) Å
β = 97.599 (2)°
*V* = 1696.35 (11) Å^3^
*Z* = 4
Mo *K*α radiation
μ = 0.20 mm^−1^
*T* = 173 K
0.29 × 0.23 × 0.17 mm

#### Data collection

Bruker SMART APEXII CCD diffractometer
Absorption correction: multi-scan (*SADABS*; Bruker, 2009 ▶) *T*_min_ = 0.653, *T*_max_ = 0.746
12919 measured reflections
2994 independent reflections
2154 reflections with *I* > 2σ(*I*)
*R*_int_ = 0.043

#### Refinement

*R*[*F* ^2^ > 2σ(*F* ^2^)] = 0.059
*wR*(*F* ^2^) = 0.173
*S* = 1.07
2994 reflections
212 parameters
14 restraints
H-atom parameters constrained
Δρ_max_ = 0.86 e Å^−3^
Δρ_min_ = −0.36 e Å^−3^

### 

Data collection: *APEX2* (Bruker, 2009 ▶); cell refinement: *SAINT* (Bruker, 2009 ▶); data reduction: *SAINT*; program(s) used to solve structure: *SHELXS97* (Sheldrick, 2008 ▶); program(s) used to refine structure: *SHELXL97* (Sheldrick, 2008 ▶); molecular graphics: *ORTEP-3* (Farrugia, 2012 ▶) and *DIAMOND* (Brandenburg, 1998 ▶); software used to prepare material for publication: *SHELXL97*.

## Supplementary Material

Crystal structure: contains datablock(s) I. DOI: 10.1107/S1600536812042249/hg5259sup1.cif

Structure factors: contains datablock(s) I. DOI: 10.1107/S1600536812042249/hg5259Isup2.hkl

Supplementary material file. DOI: 10.1107/S1600536812042249/hg5259Isup3.cml

Additional supplementary materials:  crystallographic information; 3D view; checkCIF report

## Figures and Tables

**Table 1:** Hydrogen-bond geometry (Å, °) *Cg* is the centroid of the C2–C7 benzene ring.

*D*—H⋯*A*	*D*—H	H⋯*A*	*D*⋯*A*	*D*—H⋯*A*
C20—H20*B*⋯*Cg* ^i^	0.98	2.78	3.665 (2)	150

Symmetry code: (i) 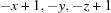.

**Fig. 1.**
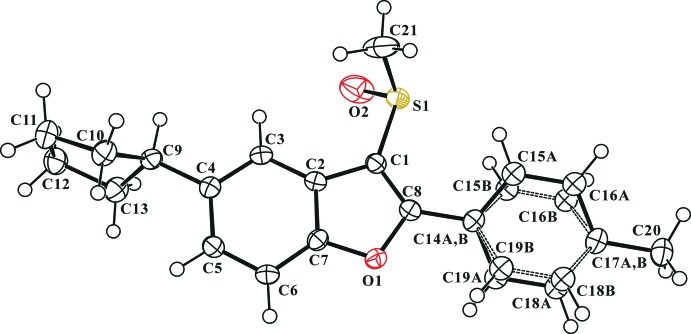
The molecular structure of the title compound with the atom numbering scheme. Displacement ellipsoids are drawn at the 50% probability level. H atoms are presented as small spheres of arbitrary radius. In the phenyl ring of the 4-methylphenyl group, the C15/C16/C18/C19 atoms are disordered over two positions with site-ccupancy factors, from refinement of 0.899 (5) (part A) and 0.101 (5) (part B).

**Fig. 2.**
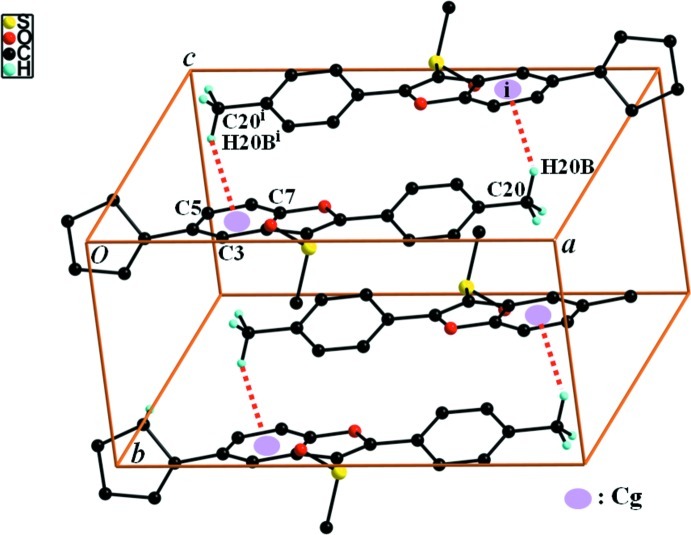
A view of C—H···π interactions (dotted lines) in the crystal structure of the title compound. H atoms non-participating in hydrogen-bonding and disordered part B atoms were omitted for clarity. [Symmetry code: (i) - x + 1, - y, - z + 1.]

## supplementary crystallographic information

### Comment

As a part of our ongoing study of 5-cyclopentyl-3-methylsulfinyl-1-benzofuran derivatives containing 2-phenyl (Choi *et al.*, 2011) and 2-(4-fluorophenyl) (Seo *et al.*,2011) substituents, we report herein the crystal structure of the title compound.

In the title molecule (Fig. 1), the benzofuran unit is essentially planar, with a mean deviation of 0.008 (2) Å from the least-squares plane defined by the nine constituent atoms. The cyclopentyl ring has an envelope conformation. In the phenyl ring of the 4-methylphenyl group, the four C atoms (C15/C16/C18/C19) are disordered over two positions with site-ccupancy factors, from refinement of of 0.899 (5) (part A) and 0.101 (5) (part B). In the crystal structure, molecules are connected via pairs of weak C—H···π interactions (Fig. 2 & Table 1, Cg is the centroid of the C2–C7 benzene ring), forming inversion dimers.

### Experimental

3-Chloroperoxybenzoic acid (77%, 224 mg, 1.0 mmol) was added in small portions to a stirred solution of 5-cyclopentyl-2-(4-methylphenyl)-3-methylsulfanyl-1-benzofuran (290 mg, 0.9 mmol) in dichloromethane (30 mL) at 273 K. After being stirred at room temperature for 5h, the mixture was washed with saturated sodium bicarbonate solution and the organic layer was separated, dried over magnesium sulfate, filtered and concentrated at reduced pressure. The residue was purified by column chromatography (hexane–ethyl acetate, 1:1 v/v) to afford the title compound as a colorless solid [yield 71%, m.p. 424–425 K; *R*f = 0.65 (hexane–ethyl acetate, 1:1 v/v)]. Single crystals suitable for X-ray diffraction were prepared by slow evaporation of a solution of the title compound in acetone at room temperature.

### Refinement

All H atoms were positioned geometrically and refined using a riding model, with C—H = 0.95 Å for aryl,1.00 Å for methine, 0.99 Å for methylene and 0.98 Å for methyl H atoms, respectively.*U*_iso_(H) = 1.2*U*_eq_(C) for aryl, methine and methylene, and 1.5*U*_eq_(C) for methyl H atoms. The positions of methyl hydrogens were optimized rotationally. In the phenyl ring of the 4-methylphenyl group, the C15/C16/C18/C19 atoms are disordered over two positions with site occupancy factors, from refinement of of 0.899 (5) (part A) and 0.101 (5) (part B). The distance of equivalent C–C pairs were restrained to 0.002 Å using the SHELXL-97 command SADI, and C14 and C17 set, was refined using EXYZ,and C14–C19 set was refined using EADP.

### Crystal data

**Table:**

C_21_H_22_O_2_S	*F*(000) = 720
*M**_r_* = 338.45	*D*_x_ = 1.325 Mg m^−^^3^
Monoclinic, *P*2_1_/*c*	Melting point = 424–425 K
Hall symbol: -P 2ybc	Mo *K*α radiation, λ = 0.71073 Å
*a* = 15.2869 (5) Å	Cell parameters from 2479 reflections
*b* = 7.2881 (3) Å	θ = 2.7–28.1°
*c* = 15.3608 (6) Å	µ = 0.20 mm^−^^1^
β = 97.599 (2)°	*T* = 173 K
*V* = 1696.35 (11) Å^3^	Block, colourless
*Z* = 4	0.29 × 0.23 × 0.17 mm

### Data collection

**Table:**

Bruker SMART APEXII CCD diffractometer	2994 independent reflections
Radiation source: rotating anode	2154 reflections with *I* > 2σ(*I*)
Graphite multilayer monochromator	*R*_int_ = 0.043
Detector resolution: 10.0 pixels mm^-1^	θ_max_ = 25.0°, θ_min_ = 2.7°
φ– and ω–scans	*h* = −18→18
Absorption correction: multi-scan (*SADABS*; Bruker, 2009)	*k* = −8→8
*T*_min_ = 0.653, *T*_max_ = 0.746	*l* = −16→18
12919 measured reflections	

### Refinement

**Table:**

Refinement on *F*^2^	Primary atom site location: structure-invariant direct methods
Least-squares matrix: full	Secondary atom site location: difference Fourier map
*R*[*F*^2^ > 2σ(*F*^2^)] = 0.059	Hydrogen site location: difference Fourier map
*wR*(*F*^2^) = 0.173	H-atom parameters constrained
*S* = 1.07	*w* = 1/[σ^2^(*F*_o_^2^) + (0.0828*P*)^2^ + 1.5926*P*] where *P* = (*F*_o_^2^ + 2*F*_c_^2^)/3
2994 reflections	(Δ/σ)_max_ < 0.001
212 parameters	Δρ_max_ = 0.86 e Å^−^^3^
14 restraints	Δρ_min_ = −0.36 e Å^−^^3^

### Special details

**Table:**

Geometry. All esds (except the esd in the dihedral angle between two l.s. planes) are estimated using the full covariance matrix. The cell esds are taken into account individually in the estimation of esds in distances, angles and torsion angles; correlations between esds in cell parameters are only used when they are defined by crystal symmetry. An approximate (isotropic) treatment of cell esds is used for estimating esds involving l.s. planes.
Refinement. Refinement of F^2^ against ALL reflections. The weighted R-factor wR and goodness of fit S are based on F^2^, conventional R-factors R are based on F, with F set to zero for negative F^2^. The threshold expression of F^2^ > 2sigma(F^2^) is used only for calculating R-factors(gt) etc. and is not relevant to the choice of reflections for refinement. R-factors based on F^2^ are statistically about twice as large as those based on F, and R- factors based on ALL data will be even larger.

### Fractional atomic coordinates and isotropic or equivalent isotropic displacement parameters (Å^2^)

**Table:**

	*x*	*y*	*z*	*U*_iso_*/*U*_eq_	Occ. (<1)
S1	0.39884 (5)	0.23817 (10)	0.27487 (5)	0.0298 (3)	
O2	0.33603 (17)	0.1030 (4)	0.22826 (15)	0.0577 (7)	
O1	0.37504 (12)	0.2498 (2)	0.52846 (12)	0.0243 (5)	
C1	0.36912 (17)	0.2579 (3)	0.38147 (18)	0.0231 (6)	
C2	0.27970 (17)	0.2531 (3)	0.40194 (18)	0.0214 (6)	
C3	0.19556 (17)	0.2513 (3)	0.35376 (18)	0.0230 (6)	
H3	0.1885	0.2516	0.2914	0.028*	
C4	0.12236 (18)	0.2493 (3)	0.39841 (19)	0.0228 (6)	
C5	0.13432 (18)	0.2478 (3)	0.49058 (18)	0.0248 (6)	
H5	0.0837	0.2471	0.5204	0.030*	
C6	0.21657 (18)	0.2473 (3)	0.53943 (18)	0.0253 (6)	
H6	0.2239	0.2459	0.6018	0.030*	
C7	0.28779 (17)	0.2491 (3)	0.49274 (18)	0.0224 (6)	
C8	0.42346 (17)	0.2554 (3)	0.45874 (18)	0.0224 (6)	
C9	0.03109 (17)	0.2503 (3)	0.34703 (19)	0.0244 (6)	
H9	0.0380	0.2472	0.2832	0.029*	
C10	−0.02605 (17)	0.4168 (4)	0.36192 (19)	0.0309 (7)	
H10A	−0.0044	0.5284	0.3349	0.037*	
H10B	−0.0269	0.4391	0.4254	0.037*	
C11	−0.11767 (18)	0.3623 (5)	0.3166 (2)	0.0388 (8)	
H11A	−0.1260	0.4038	0.2547	0.047*	
H11B	−0.1643	0.4178	0.3472	0.047*	
C12	−0.12123 (18)	0.1522 (5)	0.3216 (2)	0.0382 (8)	
H12A	−0.1347	0.0988	0.2621	0.046*	
H12B	−0.1672	0.1126	0.3573	0.046*	
C13	−0.02941 (17)	0.0915 (4)	0.3647 (2)	0.0319 (7)	
H13A	−0.0288	0.0722	0.4285	0.038*	
H13B	−0.0111	−0.0235	0.3379	0.038*	
C14A	0.51894 (17)	0.2566 (3)	0.48277 (18)	0.0232 (7)	0.899 (5)
C15A	0.57418 (15)	0.3215 (5)	0.4245 (2)	0.0300 (5)	0.899 (5)
H15A	0.5495	0.3688	0.3690	0.036*	0.899 (5)
C16A	0.66532 (18)	0.3175 (5)	0.4470 (2)	0.0300 (5)	0.899 (5)
H16A	0.7022	0.3584	0.4057	0.036*	0.899 (5)
C17A	0.70343 (17)	0.2548 (3)	0.52866 (19)	0.0262 (7)	0.899 (5)
C18A	0.64777 (15)	0.1982 (5)	0.5875 (2)	0.0300 (5)	0.899 (5)
H18A	0.6724	0.1585	0.6444	0.036*	0.899 (5)
C19A	0.55714 (18)	0.1983 (5)	0.5653 (2)	0.0300 (5)	0.899 (5)
H19A	0.5205	0.1581	0.6069	0.036*	0.899 (5)
C14B	0.51894 (17)	0.2566 (3)	0.48277 (18)	0.0232 (7)	0.10
C15B	0.5736 (12)	0.174 (4)	0.4248 (19)	0.0300 (5)	0.10
H15B	0.5489	0.1184	0.3711	0.036*	0.101 (5)
C16B	0.6644 (13)	0.178 (4)	0.4502 (10)	0.0300 (5)	0.10
H16B	0.7016	0.1261	0.4119	0.036*	0.101 (5)
C17B	0.70343 (17)	0.2548 (3)	0.52866 (19)	0.0262 (7)	0.10
C18B	0.6487 (11)	0.308 (4)	0.5890 (11)	0.0300 (5)	0.10
H18B	0.6734	0.3435	0.6465	0.036*	0.101 (5)
C19B	0.5582 (12)	0.309 (4)	0.5658 (8)	0.0300 (5)	0.10
H19B	0.5217	0.3468	0.6080	0.036*	0.101 (5)
C20	0.80136 (18)	0.2528 (4)	0.5536 (2)	0.0351 (8)	
H20A	0.8300	0.2983	0.5044	0.053*	
H20B	0.8210	0.1271	0.5678	0.053*	
H20C	0.8172	0.3317	0.6049	0.053*	
C21	0.3672 (2)	0.4541 (5)	0.2339 (2)	0.0483 (9)	
H21A	0.3689	0.4564	0.1704	0.072*	
H21B	0.4078	0.5468	0.2623	0.072*	
H21C	0.3072	0.4808	0.2460	0.072*	

### Atomic displacement parameters (Å^2^)

**Table:**

	*U*^11^	*U*^22^	*U*^33^	*U*^12^	*U*^13^	*U*^23^
S1	0.0305 (4)	0.0384 (5)	0.0214 (4)	0.0008 (3)	0.0067 (3)	−0.0013 (3)
O2	0.0737 (17)	0.0619 (17)	0.0379 (14)	−0.0226 (14)	0.0086 (12)	−0.0090 (13)
O1	0.0240 (10)	0.0286 (11)	0.0196 (10)	0.0005 (8)	0.0007 (8)	−0.0009 (8)
C1	0.0226 (13)	0.0249 (14)	0.0223 (14)	0.0016 (11)	0.0048 (11)	0.0017 (11)
C2	0.0262 (14)	0.0185 (13)	0.0201 (14)	0.0004 (10)	0.0051 (11)	−0.0009 (11)
C3	0.0275 (14)	0.0231 (14)	0.0180 (14)	0.0003 (11)	0.0020 (11)	0.0003 (11)
C4	0.0236 (14)	0.0192 (14)	0.0253 (15)	0.0000 (10)	0.0022 (11)	0.0004 (11)
C5	0.0246 (14)	0.0259 (15)	0.0243 (15)	−0.0008 (11)	0.0052 (11)	0.0002 (12)
C6	0.0293 (15)	0.0285 (15)	0.0183 (14)	0.0000 (11)	0.0035 (11)	−0.0002 (12)
C7	0.0225 (14)	0.0212 (14)	0.0227 (15)	−0.0008 (11)	−0.0002 (11)	−0.0013 (11)
C8	0.0250 (14)	0.0205 (14)	0.0224 (14)	0.0000 (11)	0.0052 (11)	−0.0015 (11)
C9	0.0249 (14)	0.0268 (15)	0.0209 (14)	−0.0005 (11)	0.0008 (11)	−0.0003 (12)
C10	0.0300 (15)	0.0300 (16)	0.0318 (17)	0.0035 (12)	0.0008 (12)	−0.0011 (13)
C11	0.0247 (16)	0.049 (2)	0.0411 (19)	0.0059 (14)	0.0006 (13)	0.0031 (16)
C12	0.0257 (16)	0.050 (2)	0.0375 (18)	−0.0083 (14)	0.0003 (13)	−0.0014 (15)
C13	0.0301 (15)	0.0291 (16)	0.0354 (17)	−0.0051 (12)	−0.0001 (13)	−0.0006 (13)
C14A	0.0244 (14)	0.0200 (14)	0.0243 (15)	0.0000 (11)	0.0002 (11)	−0.0019 (11)
C15A	0.0283 (9)	0.0332 (11)	0.0279 (9)	−0.0013 (7)	0.0020 (7)	0.0012 (8)
C16A	0.0283 (9)	0.0332 (11)	0.0279 (9)	−0.0013 (7)	0.0020 (7)	0.0012 (8)
C17A	0.0247 (14)	0.0206 (14)	0.0322 (17)	0.0009 (11)	−0.0006 (12)	−0.0041 (12)
C18A	0.0283 (9)	0.0332 (11)	0.0279 (9)	−0.0013 (7)	0.0020 (7)	0.0012 (8)
C19A	0.0283 (9)	0.0332 (11)	0.0279 (9)	−0.0013 (7)	0.0020 (7)	0.0012 (8)
C14B	0.0244 (14)	0.0200 (14)	0.0243 (15)	0.0000 (11)	0.0002 (11)	−0.0019 (11)
C15B	0.0283 (9)	0.0332 (11)	0.0279 (9)	−0.0013 (7)	0.0020 (7)	0.0012 (8)
C16B	0.0283 (9)	0.0332 (11)	0.0279 (9)	−0.0013 (7)	0.0020 (7)	0.0012 (8)
C17B	0.0247 (14)	0.0206 (14)	0.0322 (17)	0.0009 (11)	−0.0006 (12)	−0.0041 (12)
C18B	0.0283 (9)	0.0332 (11)	0.0279 (9)	−0.0013 (7)	0.0020 (7)	0.0012 (8)
C19B	0.0283 (9)	0.0332 (11)	0.0279 (9)	−0.0013 (7)	0.0020 (7)	0.0012 (8)
C20	0.0241 (15)	0.0390 (18)	0.0413 (19)	−0.0006 (13)	0.0007 (13)	0.0002 (14)
C21	0.068 (2)	0.052 (2)	0.0264 (18)	0.0205 (18)	0.0108 (16)	0.0108 (16)

### Geometric parameters (Å, º)

**Table:**

S1—O2	1.491 (2)	C12—H12A	0.9900
S1—C21	1.740 (3)	C12—H12B	0.9900
S1—C1	1.762 (3)	C13—H13A	0.9900
O1—C7	1.374 (3)	C13—H13B	0.9900
O1—C8	1.381 (3)	C14A—C19A	1.390 (4)
C1—C8	1.356 (4)	C14A—C15A	1.393 (3)
C1—C2	1.443 (4)	C15A—C16A	1.391 (3)
C2—C7	1.384 (4)	C15A—H15A	0.9500
C2—C3	1.397 (4)	C16A—C17A	1.389 (4)
C3—C4	1.388 (4)	C16A—H16A	0.9500
C3—H3	0.9500	C17A—C18A	1.384 (3)
C4—C5	1.403 (4)	C17A—C20	1.496 (4)
C4—C9	1.510 (4)	C18A—C19A	1.382 (3)
C5—C6	1.376 (4)	C18A—H18A	0.9500
C5—H5	0.9500	C19A—H19A	0.9500
C6—C7	1.381 (4)	C15B—C16B	1.392 (3)
C6—H6	0.9500	C15B—H15B	0.9500
C8—C14A	1.458 (4)	C16B—H16B	0.9500
C9—C13	1.528 (4)	C18B—C19B	1.383 (3)
C9—C10	1.529 (4)	C18B—H18B	0.9500
C9—H9	1.0000	C19B—H19B	0.9500
C10—C11	1.532 (4)	C20—H20A	0.9800
C10—H10A	0.9900	C20—H20B	0.9800
C10—H10B	0.9900	C20—H20C	0.9800
C11—C12	1.534 (5)	C21—H21A	0.9800
C11—H11A	0.9900	C21—H21B	0.9800
C11—H11B	0.9900	C21—H21C	0.9800
C12—C13	1.536 (4)		
			
O2—S1—C21	107.08 (17)	C11—C12—C13	105.8 (2)
O2—S1—C1	105.74 (13)	C11—C12—H12A	110.6
C21—S1—C1	99.72 (14)	C13—C12—H12A	110.6
C7—O1—C8	106.4 (2)	C11—C12—H12B	110.6
C8—C1—C2	107.3 (2)	C13—C12—H12B	110.6
C8—C1—S1	127.5 (2)	H12A—C12—H12B	108.7
C2—C1—S1	124.6 (2)	C9—C13—C12	104.2 (2)
C7—C2—C3	119.1 (2)	C9—C13—H13A	110.9
C7—C2—C1	105.0 (2)	C12—C13—H13A	110.9
C3—C2—C1	135.8 (3)	C9—C13—H13B	110.9
C4—C3—C2	119.0 (2)	C12—C13—H13B	110.9
C4—C3—H3	120.5	H13A—C13—H13B	108.9
C2—C3—H3	120.5	C19A—C14A—C15A	118.2 (3)
C3—C4—C5	119.5 (3)	C19A—C14A—C8	121.0 (2)
C3—C4—C9	119.4 (2)	C15A—C14A—C8	120.8 (3)
C5—C4—C9	121.0 (2)	C16A—C15A—C14A	120.4 (3)
C6—C5—C4	122.5 (3)	C16A—C15A—H15A	119.8
C6—C5—H5	118.7	C14A—C15A—H15A	119.8
C4—C5—H5	118.7	C17A—C16A—C15A	121.2 (3)
C5—C6—C7	116.3 (3)	C17A—C16A—H16A	119.4
C5—C6—H6	121.9	C15A—C16A—H16A	119.4
C7—C6—H6	121.9	C18A—C17A—C16A	117.9 (3)
O1—C7—C6	125.7 (2)	C18A—C17A—C20	120.7 (3)
O1—C7—C2	110.8 (2)	C16A—C17A—C20	121.4 (3)
C6—C7—C2	123.5 (3)	C19A—C18A—C17A	121.4 (3)
C1—C8—O1	110.5 (2)	C19A—C18A—H18A	119.3
C1—C8—C14A	134.3 (3)	C17A—C18A—H18A	119.3
O1—C8—C14A	115.2 (2)	C18A—C19A—C14A	120.8 (3)
C4—C9—C13	116.3 (2)	C18A—C19A—H19A	119.6
C4—C9—C10	115.6 (2)	C14A—C19A—H19A	119.6
C13—C9—C10	101.7 (2)	C16B—C15B—H15B	121.4
C4—C9—H9	107.6	C15B—C16B—H16B	118.3
C13—C9—H9	107.6	C19B—C18B—H18B	119.9
C10—C9—H9	107.6	C18B—C19B—H19B	119.0
C9—C10—C11	103.4 (2)	C17A—C20—H20A	109.5
C9—C10—H10A	111.1	C17A—C20—H20B	109.5
C11—C10—H10A	111.1	H20A—C20—H20B	109.5
C9—C10—H10B	111.1	C17A—C20—H20C	109.5
C11—C10—H10B	111.1	H20A—C20—H20C	109.5
H10A—C10—H10B	109.0	H20B—C20—H20C	109.5
C10—C11—C12	105.8 (2)	S1—C21—H21A	109.5
C10—C11—H11A	110.6	S1—C21—H21B	109.5
C12—C11—H11A	110.6	H21A—C21—H21B	109.5
C10—C11—H11B	110.6	S1—C21—H21C	109.5
C12—C11—H11B	110.6	H21A—C21—H21C	109.5
H11A—C11—H11B	108.7	H21B—C21—H21C	109.5
			
O2—S1—C1—C8	133.7 (3)	C7—O1—C8—C1	−0.1 (3)
C21—S1—C1—C8	−115.4 (3)	C7—O1—C8—C14A	−179.9 (2)
O2—S1—C1—C2	−36.8 (3)	C3—C4—C9—C13	123.0 (3)
C21—S1—C1—C2	74.2 (3)	C5—C4—C9—C13	−57.6 (3)
C8—C1—C2—C7	0.4 (3)	C3—C4—C9—C10	−117.8 (3)
S1—C1—C2—C7	172.45 (19)	C5—C4—C9—C10	61.6 (3)
C8—C1—C2—C3	−179.4 (3)	C4—C9—C10—C11	−169.5 (2)
S1—C1—C2—C3	−7.3 (4)	C13—C9—C10—C11	−42.5 (3)
C7—C2—C3—C4	1.3 (4)	C9—C10—C11—C12	28.5 (3)
C1—C2—C3—C4	−179.0 (3)	C10—C11—C12—C13	−3.3 (3)
C2—C3—C4—C5	−0.4 (4)	C4—C9—C13—C12	167.1 (2)
C2—C3—C4—C9	179.1 (2)	C10—C9—C13—C12	40.5 (3)
C3—C4—C5—C6	−0.4 (4)	C11—C12—C13—C9	−23.1 (3)
C9—C4—C5—C6	−179.8 (2)	C1—C8—C14A—C19A	−160.3 (3)
C4—C5—C6—C7	0.2 (4)	O1—C8—C14A—C19A	19.4 (4)
C8—O1—C7—C6	−178.8 (2)	C1—C8—C14A—C15A	21.6 (4)
C8—O1—C7—C2	0.3 (3)	O1—C8—C14A—C15A	−158.7 (3)
C5—C6—C7—O1	179.7 (2)	C19A—C14A—C15A—C16A	3.6 (4)
C5—C6—C7—C2	0.7 (4)	C8—C14A—C15A—C16A	−178.2 (3)
C3—C2—C7—O1	179.4 (2)	C14A—C15A—C16A—C17A	−2.1 (5)
C1—C2—C7—O1	−0.4 (3)	C15A—C16A—C17A—C18A	−0.7 (5)
C3—C2—C7—C6	−1.5 (4)	C15A—C16A—C17A—C20	−179.1 (3)
C1—C2—C7—C6	178.7 (2)	C16A—C17A—C18A—C19A	2.0 (5)
C2—C1—C8—O1	−0.2 (3)	C20—C17A—C18A—C19A	−179.6 (3)
S1—C1—C8—O1	−171.98 (18)	C17A—C18A—C19A—C14A	−0.4 (5)
C2—C1—C8—C14A	179.6 (3)	C15A—C14A—C19A—C18A	−2.4 (5)
S1—C1—C8—C14A	7.8 (4)	C8—C14A—C19A—C18A	179.4 (3)

### Hydrogen-bond geometry (Å, º)

Cg is the centroid of the C2–C7 benzene ring.

**Table:**

*D*—H···*A*	*D*—H	H···*A*	*D*···*A*	*D*—H···*A*
C20—H20*B*···*Cg*^i^	0.98	2.78	3.665 (2)	150

Symmetry code: (i) −*x*+1, −*y*, −*z*+1.
